# Pubic Pediculosis

**DOI:** 10.4269/ajtmh.22-0265

**Published:** 2022-07-18

**Authors:** Xiujiao Xia, Zehu Liu

**Affiliations:** Department of Dermatology, Hangzhou Third People’s Hospital, Affiliated Hangzhou Dermatology Hospital, Zhejiang University School of Medicine, Hangzhou, People’s Republic of China

**Figure 1.**
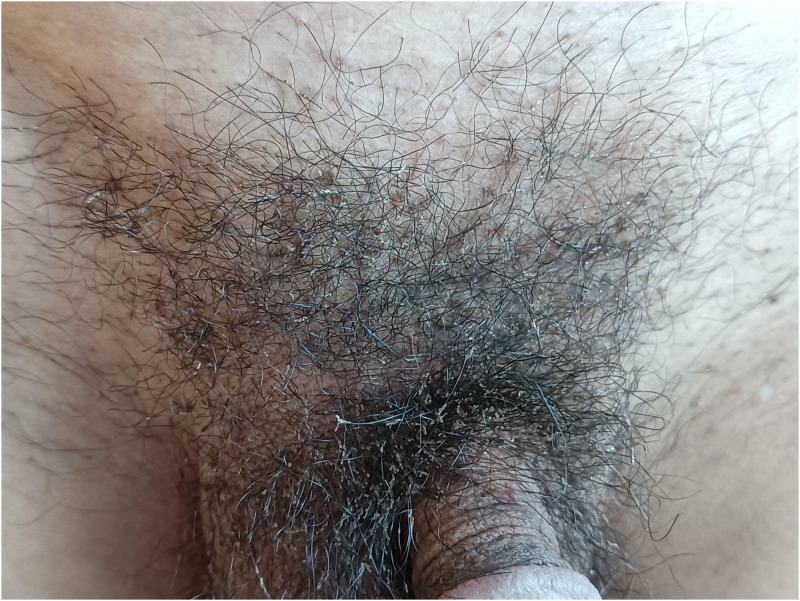
Multiple black dots on the base of the pubic hair shaft and whitish-appearing concretions attached to the shafts of pubic hairs. This figure appears in color at www.ajtmh.org.

**Figure 2.**
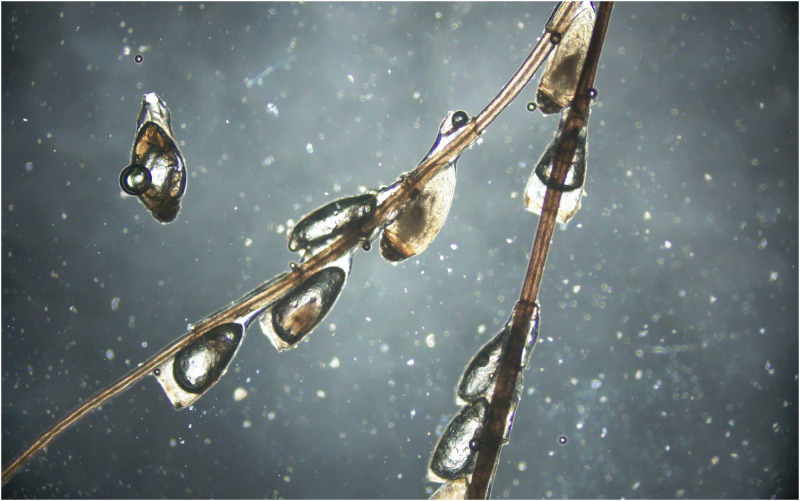
An array of lice eggs attached to the shafts of pubic hairs under direct microscopic examination (magnification ×40). This figure appears in color at www.ajtmh.org.

A 65-year-old man presented to the dermatology clinic with a 6-month history of severe itching in the inguinal area on February 25, 2022. This patient worked at a construction site and reported that he had had no recent sexual contact. He had treated himself with diesel oil, but there was no improvement in his condition. Physical examination was notable for multiple black dots on the base of the pubic hair shaft and whitish-appearing concretions attached to the shafts of pubic hairs (Figure 1), microscopic examination of the hair sample showed an array of insect eggs like silkworm chrysalis attached to the shafts of pubic hairs (Figure 2).

There are more than 3,000 species of lice. Among these, humans constitute the preferred host for only two species: *Pediculus humanus* and *Phthirus pubis* (pubic lice).^1^ Once the pubic lice make its headquarters in the pubic hair, the lice life cycle begins. A female lice lays approximately eight eggs a day, and can lay up to 300 eggs during her lifetime. Eggs, or, nits, laid by the female adult are securely cemented to hairs.^2^ Finally, this patient was treated successfully with shaving of the abdomen and pubic area and with compound sulfur cream.
